# JAG2/Notch2 inhibits intervertebral disc degeneration by modulating cell proliferation, apoptosis, and extracellular matrix

**DOI:** 10.1186/s13075-019-1990-z

**Published:** 2019-10-16

**Authors:** Jun Long, Xiaobo Wang, Xianfa Du, Hehai Pan, Jianru Wang, Zemin Li, Hui Liu, Xudong Li, Zhaomin Zheng

**Affiliations:** 1 Department of Spine Surgery, First Affiliated Hospital of Sun Yat-sen University, 58 Zhongshan Second Road, Guangzhou, People’s Republic of China; 2 Organ Transplant Center, The First Affiliated Hospital of Sun Yat-sen University, 58 Zhongshan Second Road, Guangzhou, People’s Republic of China; 3 Pain Research Center and Department of Physiology, Zhongshan Medical School of Sun Yat-sen University, 74 Zhongshan Second Road, Guangzhou, People’s Republic of China

**Keywords:** Low back pain, Intervertebral disc degeneration, Nucleus pulposus cell, Notch signaling

## Abstract

**Background:**

Intervertebral disc degeneration (IVDD)-related disorders are the major causes of low back pain. A previous study suggested that Notch activation serves as a protective mechanism and is a part of the compensatory response that maintains the necessary resident nucleus pulposus (NP) cell proliferation to replace lost or non-functional cells. However, the exact mechanism remains to be determined. In this study, we aimed to investigate the role of JAG2/Notch2 in NP cell proliferation and apoptosis.

**Methods:**

Recombinant JAG2 or Notch2, Hes1, and Hey2 siRNAs were used to activate or inhibit Notch signaling. Cell proliferation, apoptosis, cell cycle regulatory factors, and pathways associated with Notch-mediated proliferation were examined. In vivo experiments involving an intradiscal injection of Sprague-Dawley rats were performed.

**Results:**

Recombinant JAG2 induced Notch2 and Hes1/Hey2 expression together with NP cell proliferation. Downregulation of Notch2/Hes1/Hey2 induced G0/G1 phase cell cycle arrest in NP cells. Moreover, Notch2 mediated NP cell proliferation by regulating cyclin D1 and by activating PI3K/Akt and Wnt/β-catenin signaling. Furthermore, Notch signaling inhibited TNF-α-promoted NP cell apoptosis by suppressing the formation of the RIP1-FADD-caspase-8 complex. Finally, we found that intradiscal injection of JAG2 alleviated IVDD and that sh-Notch2 aggravated IVDD in a rat model. These results indicated that JAG2/Notch2 inhibited IVDD by modulating cell proliferation, apoptosis, and extracellular matrix. The JAG2/Notch2 axis regulated NP cell proliferation via PI3K/Akt and Wnt/β-catenin signaling and inhibited TNF-α-induced apoptosis by suppressing the formation of the RIP1-FADD-caspase-8 complex.

**Conclusions:**

The current and previous results shed light on the therapeutic implications of targeting the JAG2/Notch2 axis to inhibit or reverse IVDD.

## Introduction

Low back pain (LBP) is one of the most common and expensive health problems, affecting about 80% of the population during their life with total costs exceeding $100 billion per year [1, 2]. Intervertebral disc degeneration (IVDD)-related disorders are the major causes of LBP and associated disability [3]. The hallmarks of IVDD are decreased cell density [4] and extracellular matrix (ECM) [5], increased fibrosis, increased cell death, and a transition from notochordal cells to chondrocyte-like cells that exhibit the characteristics of hypertrophic chondrocytes [6, 7]. Nucleus pulposus (NP) cells that produce the proteoglycan aggrecan and collagens to form complex ECM play key roles in maintaining healthy discs [8]. Many studies have found that cytokines mediate the shift in NP cell function and promote abnormal proliferation and apoptosis [9, 10]. Apoptosis of NP cells has also been reported as one of the initial triggers of IVDD [11]. Various factors, including aging, genetics, nutrition, metabolic factors, infection, mechanical factors, signaling networks, and inflammatory cytokines, induce NP cell apoptosis, which results in progressive IVDD [12, 13]. However, the potential contribution of these factors and signaling pathways remains to be elucidated.

Notch is a highly conserved pathway [14] and is involved in multiple cellular processes, including cell fate determination, differentiation, proliferation, apoptosis, and regeneration [15]. Several studies have reported that Notch signaling plays an important role during chondrogenesis and cartilage development [16, 17]. A previous study has shown that Notch signaling is critical for the maintenance of NP cell proliferation in IVD cells [18]. Moreover, our studies suggested that Notch activation serves as a protective mechanism and is part of a compensatory response that maintains the necessary resident NP cell proliferation to replace dead or non-functional cells [19]. However, the role of Notch signaling in NP proliferation and apoptosis remains unclear. Furthermore, our previous studies only focused on an in vitro NP cellular model, and because the human body is more complex than a cellular model, in vivo experiments were performed on rats in this study.

In this study, we aimed to investigate the role of JAG2/Notch2 in IVDD, NP cell proliferation, and apoptosis, as well as the related signaling pathways. Our results showed that JAG2/Notch2 inhibited IVDD by modulating cell proliferation, apoptosis, and extracellular matrix. The current and previous results shed light on the therapeutic implications of targeting the JAG2/Notch2 axis to inhibit or reverse IVDD.

## Methods

### Collection and grading of human disc samples

From October 2014 to October 2017, four non-degenerated (grade I/II) samples from patients with spinal fracture, four mildly degenerated (grade III) samples, four moderately degenerated (grade IV) samples, and four severely degenerated (grade V) samples were collected. Normal and degenerated human lumbar IVDs were classified according to our previous description using the Pfirrmann grading scheme [4]. Each patient provided informed consent for the study, which was approved by the Ethics Committee of our institution (First Affiliated Hospital of Sun Yat-sen University).

### Isolation of NP cells and treatment

Animal experiments were performed according to the National Institutes of Health Guide for the Care and Use of Laboratory Animals. The study protocols were approved by the Animal Experimentation Committee of our institution (First Affiliated Hospital of Sun Yat-sen University).

Sprague-Dawley rats (4–6 weeks old) were euthanized by administering chloral hydrate anesthesia. The spinal columns were removed under aseptic conditions. Lumbar intervertebral discs were separated from the spinal column, while the gel-like NP tissues were separated from the discs. The NP tissues were cut into pieces, partially digested, and then maintained in Dulbecco’s modified Eagle’s medium (DMEM, Gibco, US Origin) supplemented with 10% fetal bovine serum (FBS, Gibco, US Origin) and 2% penicillin-streptomycin antibiotics in a humidified atmosphere containing 5% CO_2_ at 37 °C. One week later, the NP cells migrated out of the explant and were isolated using trypsin (0.25%) EDTA (1 mM) solution. NP cells were sub-cultured in 10-cm dishes and treated with TNF-α (100 ng/mL, Peprotech, NJ) for 24 h.

### RNA isolation and qRT-PCR

Total RNA was extracted using TRIzol (Invitrogen, Carlsbad, CA); 2 μg of total DNA-free RNA was used to synthesize cDNA with SuperScript III cDNA synthesis kit (Invitrogen, Carlsbad, CA). Reactions were set up to triplicate in 96-well plates using 1 μL cDNA with PrimeScript™ RT-PCR Kit (Takara). Each set of samples included a template-free control.

The real-time quantitative reverse transcription polymerase chain reaction (qRT-PCR) was achieved using the Taq qPCR Master Mix (Promega) on real-time PCR System following the manufacturer’s instructions. Primers were designed and synthesized by Sangon Biotech. The primers used were shown in Table 1. β-Actin were used to normalize the mRNA expression. Melting curves were analyzed to verify the specificity.

**Table 1:** Primer sequences for related genes

Gene	Primer Sequences (5′–3′)
Rat NOTCH1	Forward: ACGCCTACCTCTGCTTCTGC Reverse: CAGGCACACTCGTAGCCATC
Rat NOTCH2	Forward: ATGTGTCAACGGCTGGAGTG Reverse: GCAAGAGAAGGAGGCCACAC
Rat NOTCH3	Forward: GCGAGGCAGACATCAATGAG Reverse: ACAGCGAGGACCTGAGAAGC
Rat NOTCH4	Forward: GAAGGCCACAGACACAGCAG Reverse: CACATGACCACTCCGTCCTC
Rat JAG1	Forward: CTGTGGCTTGGATCTGTTGC Reverse: CGTTGTTGGTGGTGTTGTCC
Rat JAG2	Forward: ATCTGTGAGGACCTGGTGGATGG Reverse: GTAGCAGCGAGCACCGTTGAG
Rat delta-like1	Forward: AGCCTCCGCCTGATCCTTGC Reverse: TCCGCCTTCTTGTTGGTGTTCTTG
Rat delta-like 3	Forward: CTGCCTTGTCGTTGCCTGATGG Reverse: GCCACACGCGCTAATAGGTTCC
Rat delta-like 4	Forward: AAGCATTACCAGGCAACCTTCTCC Reverse: TTACGACCACTGCCGCTATTCTTG
Rat Hes1	Forward: CCAAGCTGGAGAAGGCAGACATTC Reverse: GGTCACCTCGTTCATGCACTCG
Rat Hes5	Forward: GACCGCATCAACAGCAGCATTG Reverse: TCTCCAGGATGTCGGCCTTCTC
Rat Hey1	Forward: CTGTCGCCGCCATGCTTCTC Reverse: GCTGCCTGTGAGGTGTCAAGAC
Rat Hey2	Forward: CTAGAGAGGACCTGGAGAGTTTAAG Reverse: CTGTGCCACCAGCCTTAAAACC
Rat CBF1	Forward: GGTGTGAGGAGGAGGAACAATGAC Reverse: GCACGAGCAGCCATCTCAGC
Rat cyclin D1	Forward: CCTGACACCAATCTCCTCAACGAC Reverse: TTCCGCATGGATGGCACAATCTC
Rat CDK6	Forward: CTCTGAAGCGCGTGCGAGTG Reverse: AAGGTCTCCAGGTGCCTCAGC
Rat p21	Forward: GTTCCTTGTGGAGCCGGAGC Reverse: GGTACAAGACAGTAGCAGATC
Rat PARP	Forward: CTAAGTGTTCGTCTTTAG Reverse: AGAAATTGTTAGCGTTCC
Rat PI3K	Forward: CAATGATGCTTGGCTCTGGAATGC Reverse: TTGTCCAGCCACCATGATGTGC
Rat Fas	Forward: GGCGGGTTCGTGAAACTGAT Reverse: AGGTTGGCATGGTTGACAGC
Rat RIP	Forward: AGCCTGGTTCACTGCACAGTTC Reverse: ATGGTAGTTGGCTTCGTCTTGGAG
Rat β-tubulin	Forward: CAGCGATGAGCACGGCATAGAC Reverse: CCAGGTTCCAAGTCCACCAGAATG
Rat β-actin	Forward: CGTCCGTGACATCAAGGAGAAGC Reverse: ACCGAGGAAGGAAGGCTGGAAG

### Protein extraction and Western blotting

Cells were placed on ice immediately following treatment. They were lifted, washed with ice-cold PBS, and harvested in mammalian protein extraction reagent buffer (Pierce, Rockford, IL). Total cell proteins were resolved on 8–12% SDS-polyacrylamide gels and transferred by electroblotting to the PVDF membranes (Bio-Rad). The membranes were blocked with 5% non-fat dry milk in TBST and incubated overnight at 4 °C in 3% non-fat dry milk in TBST with the antibodies against JAG1 (Abcam, ab109627; 1:2000 dilution), JAG2 (Abcam, ab109627; 1:2000 dilution), Notch1 (CST, #3608; 1:2000 dilution), Notch2 (CST, #5732; 1:2000 dilution), Notch3 (CST, #5276; 1:2000 dilution), Hes1 (CST, #11988; 1:2000 dilution), Hes5 (CST, #11988; 1:2000 dilution), Hey1 (Proteintech, 10597-1-AP; 1:2000 dilution), Hey2 (Proteintech, 10597-1-AP; 1:2000 dilution), cleaved caspase-3 (CST, #9661; 1:1000 dilution), caspase-8 (CST, #4790; 1:1000 dilution), caspase-9 (CST, #9508, 1:1000), anti-cyclin D1 antibody (Abcam, ab134175; 1:2000 dilution), anti-CDK6 antibody (Abcam, ab151247; 1:2000 dilution), anti-p21 antibody (Abcam, ab109199; 1:2000 dilution), phospho-Akt (CST, #4060; 1:2000 dilution), PI3K (CST, #4255; 1:2000 dilution), β-catenin (CST, #9562; 1:2000 dilution), MMP-3 (Abcam, #53015, 1:1000), MMP-13 (Abcam, #39012, 1:3000), ADAMTS-4 (Abcam, #185722, 1:1000), ADAMTS-5 (Abcam, #41037, 1:250), collagen II (Abcam, #34712, 1:5000), and aggrecan (Abcam, #3773, 1:100). β-Tubulin was from the Developmental Studies Hybridoma Bank and GAPDH was from Novus Biologicals. Immunolabeling was detected using the ECL reagent (Amersham Biosciences).

### Transfections and dual luciferase assay

Rat NP cells were transferred to 48-well plates (2 × 10^4^ cells/well) 1 day before transfection. To measure the effect of Notch signaling on cyclin D1, cells were transfected with 250 ng of reporter plasmids with 250-ng pRL-TK plasmid. Twenty-four hours after transfection, cells were treated recombinant JAG2 protein with or without Notch2/Hes1/Hey2 siRNA. Forty-eight hours after transfection, the cells were harvested and a Dual-Luciferase™ reporter assay system (Promega) was used for sequential measurements of firefly and Renilla luciferase activities. Quantification of luciferase activities and calculation of relative ratios were carried out using a luminometer (TD-20/20, Turner Designs, CA). At least three independent transfections were performed, and all analyses were carried out in triplicate.

### Cell proliferation by Cell Counting Kit-8 assay

The cell viability detection was performed according to the Cell Counting Kit-8 (CCK-8) (BD Pharmingen, USA) method [20]. Cells were seeded in 200-μL medium, and the cell density was adjusted to 5 × 10^3^ cells per well in 96-well plate. One hundred microliters of culture medium was pipetted into the wells of 96-well plates as the blank control group. The plates were kept in an incubator at 37 °C under 5% CO_2_ for 24 h. Briefly, after the treatment, 10 μL of CCK-8 (Kumamoto, Japan) was added to each well and the plates were incubated for 1 h. The optical density (OD) was measured by microplate reader (Tecan; Spectra Flour Plus) at 450 nm. The procedure was carried out thrice to obtain the mean of the collected readings. The percentage of inhibition of proliferation was calculated by (1 − mean OD for drug group/mean OD for control group) × 100%.

### Cell cycle analysis

To investigate the impact of Notch on NP cell proliferation, we evaluated the effect of Notch on progression of cell cycle. NP cells, which were in the exponential phase of growth, were treated with recombinant JAG2, with or without Notch2/Hes1/Hey2 siRNA for 36 h. Subsequently, the cell was stained with propidium iodide to determine the cell cycle distribution using flow cytometry (FACSCalibur system, BD Bioscience, USA).

### Apoptosis detection by the terminal deoxynucleotidyl transferase (TdT)-mediated dUTP nick end labeling

Terminal deoxynucleotidyl transferase (TdT)-mediated dUTP nick end labeling (TUNEL) assay (BD Pharmingen, USA) was used to measure DNA fragmentation [4]. Briefly, the cells were placed on autoclaved glass coverslips in 6-well culture plates and treated with TNF-α, or recombinant JAG2, with or without Notch2/Hes1/Hey2 siRNA. Cellular DNA was stained with apoptosis detection kits (Millipore), and the assay was performed according to the recommendations from the manufacturer.

### Flow cytometric analysis for cell apoptosis

NP cells were trypsinized and collected for the detection of apoptosis by using Annexin V-FITC Apoptosis Detection Kit (BD), according to the instructions of the manufacturer. Briefly, NP cells were detached from culture plates using 0.25% trypsin/EDTA (Invitrogen). Apoptotic NP cells were identified by staining with FITC-Annexin V/PI (BD Biosciences, San Diego, CA, USA) and then analyzed by flow cytometric (FCM). After washing, PI and FITC-Annexin V were added, and the cells were incubated at room temperature for 10 min before analysis. The index for apoptosis was calculated as the number of cells undergoing apoptosis to the number of total cells.

### IVDD model and situ apoptosis detection

A total of 32 male Sprague-Dawley rats weighing 250–300 g, aged 3 months, were used for the in vivo experiments at the center for animal experiments of Sun Yat-sen University. A model of anterior disc puncture IVD degeneration established in our previous study was adopted [21, 22]. These rats were randomly divided into four groups: non-puncture group, punctured group (control group), JAG2 group which was punctured and injected recombinant JAG2, and sh-Notch2 group which was punctured and injected lentiviral sh-Notch2. The L4/5 IVD was exposed, and a 21-gauge needle needle was inserted parallel to the endplates.

In the punctured + recombinant JAG2 injection group, 2 μL (1.6 ng) of recombinant JAG2 was slowly injected into the punctured IVD through an anterior approach using a microliter syringe (10 μL, Gaoge, Shanghai, China) 1 week post-surgery. The same procedure was repeated 2 weeks post-surgery.

In the punctured + lentiviral sh-Notch2 injection group, 2 μL of lentiviral sh-Notch2 was slowly injected into the punctured IVD through an anterior approach using a microliter syringe (10 μL, Gaoge, Shanghai, China) 1 week post-surgery. The same procedure was repeated 2 weeks post-surgery.

Lumbar MRI examinations were performed 0, 3, and 9 weeks after surgery. The Pfirrmann classification was used to assess the degree of IVD degeneration [23].

The rats were euthanized, and the L4/5 IVDs were excised along with the adjacent vertebrae. The samples were stained with TUNEL staining. The histological staining scores were graded according to the criteria established by Masuda et al. [24].

### Statistical analysis

The measurement data were presented as means ± standard deviation (SD), and a *P* value < 0.05 was considered statistically significant. Differences between the groups were estimated using Student’s *t* test and analysis of variance (ANOVA). Spearman’s correlation test was applied to assess the correlation between JAG2 and Notch2 expression. All statistical analyses were carried out by SPSS software (V19.0; SPSS, Inc., Chicago, IL, USA).

## Results

### TNF-α increases Notch ligand expression in NP cells

**Fig. 1:**
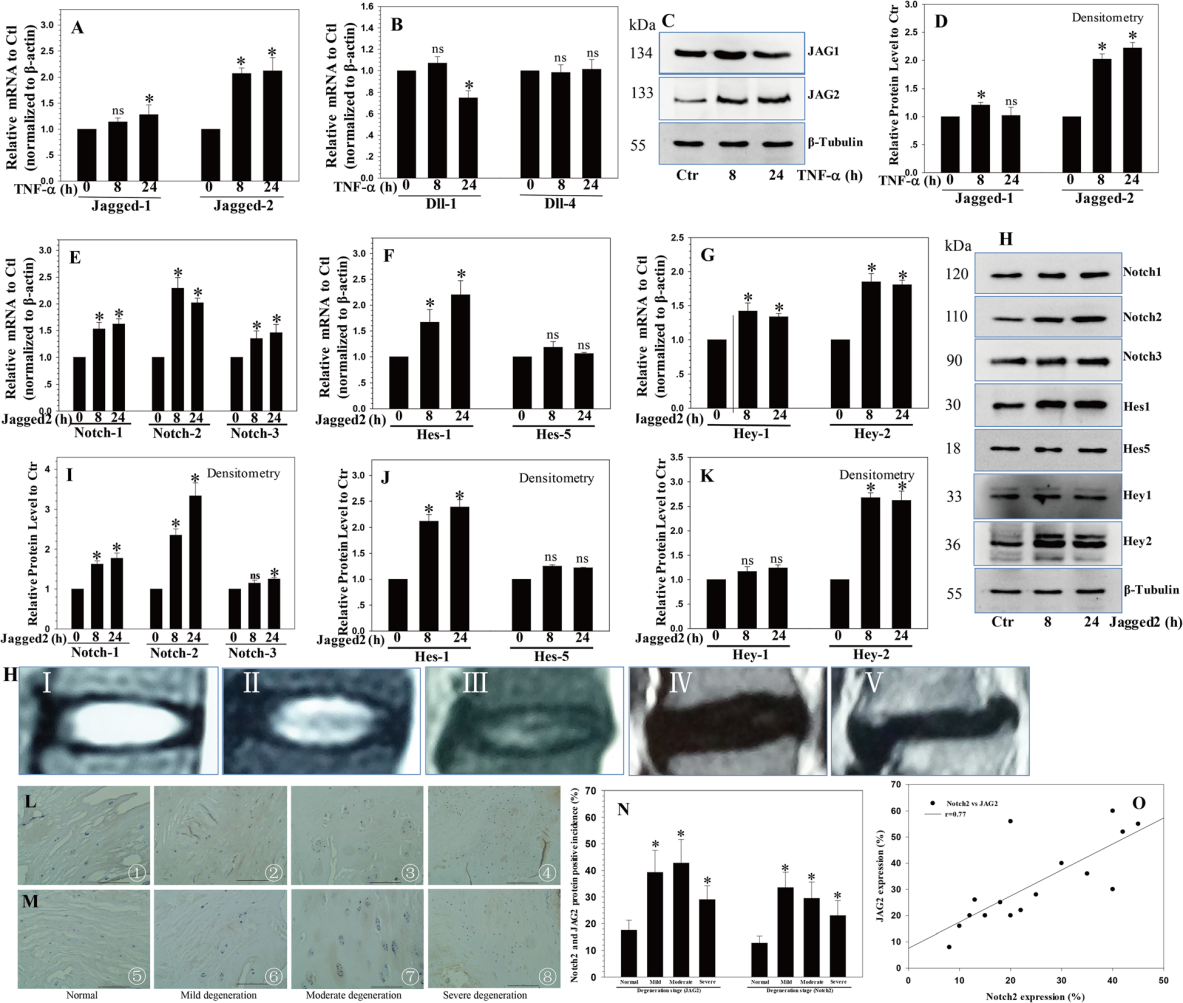
The expression of Notch-2 and Hey-2/Hes1 induced by JAG2. **a**, **b** The expression of JAG2 mRNA increased following TNF-α treatment. **c**, **d** Western blot and densitometric analyses showed similar results. **e**–**g** The expression changes in Notch-1, Notch-2, and Notch-3 mRNA and the Notch target genes Hes1/5 and Hey1/2 mRNA were regulated by the JAG2 treatment. **h**–**k** Western blot and densitometric analyses showed similar results. **h** Representative MRI images of different degenerative discs (from left, grades I, II, III, IV, and V). IHC showed that the expression of JAG2 (**j**) and Notch-2 (**k**) increased with the severity of IVD degeneration, with significantly higher positive incidences in mild and moderately degenerated IVDs (**l**). **m**–**o** Correlation analysis revealed that the expression levels of JAG2 and Notch-2 were significantly correlated. *P* values were computed vs. non-stimulated controls*; *P < 0.05. ns, no signifcant.

The results showed that TNF-α treatment increased the expression of JAG2 mRNA (Fig. 1a) and protein (Fig. 1c, d), whereas there was little change in the expression of JAG1 and Dll4 (Fig. 1a, b); moreover, the expression of Dll1 was suppressed by TNF-α (Fig. 1b). Therefore, we decided to use JAG2 for further analyses.

### Notch-2 and Hey-2/Hes1 expression is induced by JAG2

To define the role of JAG2 in regulating Notch activation and its specific receptor or target gene, we treated NP cells with recombinant JAG2. First, the expression levels of Notch-1, Notch-2, and Notch-3 were assessed. Notch-2 expression increased significantly by about 2.3-fold (*P* < 0.0001) (Fig. 1e). Notch-1 and Notch-3 expression also increased, but not as much as Notch-2 (Fig. 1e). Next, the Notch target genes Hes1/5 and Hey1/2 were analyzed. The results indicated a significant increase in the expression of Hes1 (2.2-fold), Hes5 (1.1-fold), Hey1 (1.3-fold), and Hey2 (1.9-fold), demonstrating the activation of Notch signaling in response to JAG2 treatment (all *P* < 0.05) (Fig. 1f, g). Western blot and densitometry analyses showed similar results (Fig. 1h–k).

### Elevated JAG2 and Notch2 in degenerated IVDs

Normal and degenerated human lumbar IVDs were collected according to our previously described method, with four non-degenerated (grade I/II) samples, four mildly degenerated (grade III) samples, four moderately degenerated (grade IV) samples, and four severely degenerated (grade V) samples. To determine the involvement of JAG2 and Notch-2 in IVD degeneration, immunohistochemistry (IHC) staining was performed using anti-JAG2 or anti-Notch-2. The percentages of the JAG2- or Notch-2-positive incidences increased with the severity of IVD degeneration, with significantly higher positive incidences in mild and moderately degenerated IVDs (Fig. 1j–l and Additional files 1 and 2). Importantly, the expression levels of JAG2 and Notch-2 were significantly correlated (all *P* < 0.05) (Fig. 1m).

### JAG2 induces proliferation of NP cells

**Fig. 2:**
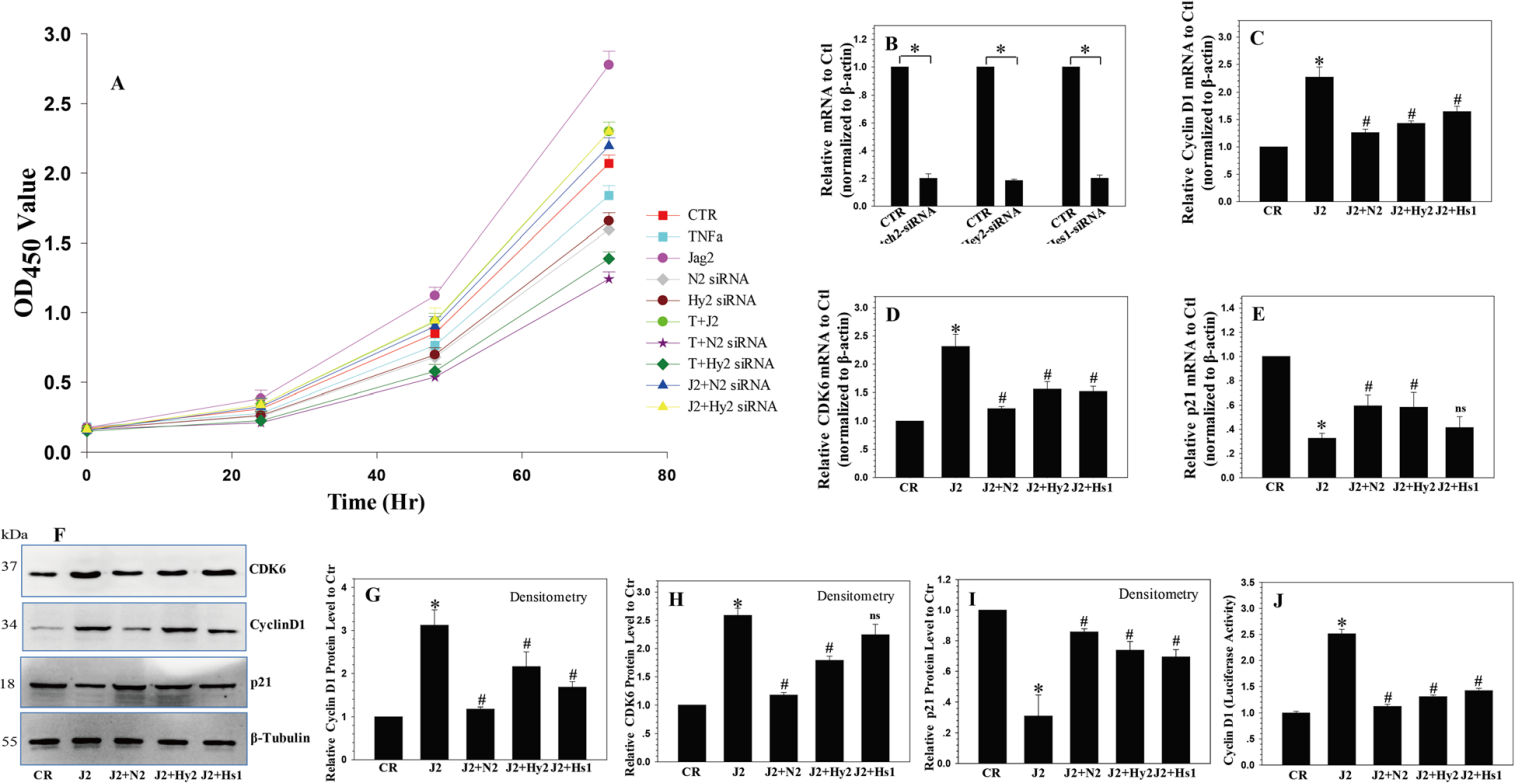
The regulating role of Notch signaling in NP cell proliferation. **a** JAG2 induced the proliferation of NP cells, and depletion of Notch2/Hey2 reduced NP cell proliferation. **b** The efficiency of the Notch2/Hey2/Hes1 siRNAs. **c**, **d** Cyclin D1 and CDK6 mRNA and protein expression levels were increased by recombinant JAG2 treatment and decreased by silencing of Notch2, Hes1, or Hey2. **f**–**h** Cyclin D1 and CDK6 protein expression levels were increased by recombinant JAG2 treatment and decreased by silencing of Notch2, Hes1, or Hey2. **e**–**i** p21 mRNA and protein expression levels were decreased by recombinant JAG2 treatment and increased by silencing of Notch2, Hes1, or Hey2. **j** Luciferase reporter assays confirmed that the activity of the cyclin D1 promoter was significantly upregulated by recombinant JAG2 treatment and downregulated by Notch2/Hey2/Hes1 siRNA. *P* values were computed vs. non-stimulated controls* or JAG2-stimulated controls^#^; *^,#^*P* < 0.05

The correlation between JAG2 expression and NP cell proliferation was analyzed using recombinant JAG2 protein treatment [25]. The CCK-8 test results revealed that JAG2 treatment caused the upregulation of NP cell proliferation (Fig. 2a).

### Depletion of Notch2/Hey2 reduces the proliferation of NP cells

To determine whether Notch2/Hey2/Hes1 expression contributes to NP cell growth, we examined the effect of the siRNA-mediated depletion of Notch2/Hey2/Hes1 together with recombinant JAG2 protein treatment on the proliferation of NP cells. NP cells were placed in the CR, TNF-α, recombinant JAG2, Notch2/Hey2 siRNA, TNF-α + JAG2, TNF-α + Notch2 siRNA, TNF-α + Hey2 siRNA, JAG2 + Notch2 siRNA, and JAG2 + Hey2 siRNA groups. Chemically synthesized siRNAs (Notch2/Hey2/Hes1) were used to treat NP cells, and the immunofluorescence detection of red fluorescent protein (RFP) in NP cells transduced with siRNA co-expressing RFP revealed a high transduction efficiency (Additional file 3). Real-time PCR was used to analyze the effects of Notch2/Hey2/Hes1 siRNA (Fig. 2b). Compared to the controls, NP cells treated with Notch2/Hey2 siRNA exhibited a significant reduction in proliferation as determined by the CCK-8 assay, with most significant reduction in Notch2 siRNA; TNF-α suppressed the proliferation of NP cells, and Notch2/Hey2 siRNA could enhance the suppression of NP cell proliferation by TNF-α; JAG2 treatment unregulated the proliferation of NP cells, while Notch2/Hey2 siRNA could offset some proliferation promotion of NP cells by JAG2 treatment (Fig. 2a).

### Cyclin D1 is critical for the Notch-mediated effect on proliferation

To further explore the associated molecular mechanisms, we focused on several known G0/G1 phase cell cycle regulatory factors. Consistent with cell cycle regulation, cyclin D1 and CDK6 mRNA and protein expression were upregulated by recombinant JAG2 treatment and downregulated by silencing of Notch2, Hes1, or Hey2, while p21 protein expression was decreased by recombinant JAG2 treatment and increased by silencing of Notch2, Hes1, or Hey2 (Fig. 2c–i). Based on previous studies that identified cyclin D1 as a Notch target in Notch-mediated proliferation in different cells [26, 27], we chose cyclin D1 for further validation.

To determine whether the activation of cyclin D1 by Notch signaling in NP cells was involved in the regulation at the transcriptional level, we tested whether the full-length cyclin D1 reporter promoter was responsive to Notch activation. The results showed that the cyclin D1 reporter plasmid was upregulated by recombinant JAG2 treatment (Fig. 2j), while Notch2/Hey2/Hes1 siRNA decreased the activity of the cyclin D1 promoter-luciferase reporter. These data demonstrated that the regulation of NP cell proliferation by the JAG2/Notch2/Hey2 axis occurred via cyclin D1.

### Downregulation of JAG2/Notch2 induces G0/G1 phase cell cycle arrest in NP cells

**Fig. 3:**
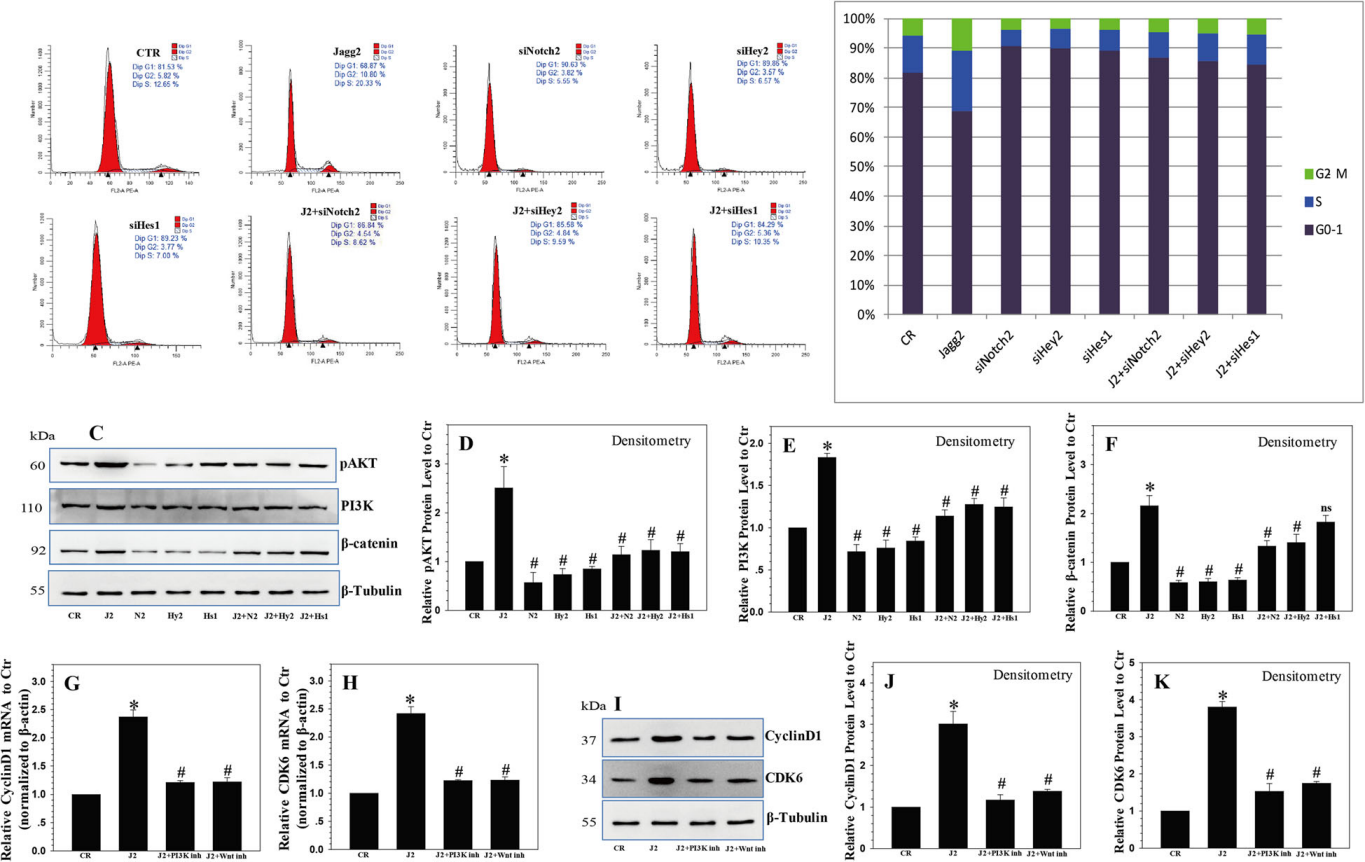
Notch-mediated proliferative effect requires functional PI3K/Akt and Wnt/β-catenin signaling. **a**, **b** Downregulation of Notch2/Hey2/Hes1 expression by siRNA led to a marked arrest in cell cycle progression, as characterized by an accumulation of NP cells in G0/G1 cells. **c**–**f** JAG2 treatment increased the expression of PI3K/Akt and Wnt/β-catenin signaling, which was inhibited by Notch2/Heys2/Hes1 siRNA. **g**, **h** The increases in cyclin D1 and CDK6 mRNA levels after JAG2 treatment were abrogated by treatment with LY294002 and XAV-939 alone or in combination. **i**–**k** Western blot and densitometric analyses showed similar results. *P* values were computed vs. non-stimulated controls* or JAG2-stimulated controls^#^; *^,#^*P* < 0.05

As cell proliferation is closely related to cell cycle progression, we analyzed the effects of the JAG2/Notch2 axis on cell cycle distribution. The results showed that cell cycle progression was promoted by recombinant JAG2 treatment. However, the downregulation of Notch2/Hey2/Hes1 expression by siRNA led to a marked arrest in cell cycle progression, characterized by the accumulation of cells in G0/G1 phase (Fig. 3a, b). These data indicate that the JAG2/Notch2 axis controls the G1 to S cell cycle progression in NP cells. Although silencing of Notch2, Hes1, or Hey2 inhibited cell cycle progression in NP cells, it did not abolish the effect of recombinant JAG2, which means there are other Notch receptors taking part in the regulation of cell cycle progression in NP cells.

### The Notch-mediated proliferative effect requires functional PI3K/Akt and Wnt/β-catenin signaling

As Notch signaling extensively cross-talks with other signaling pathways [28], we determined whether other signaling pathways were involved in the regulation of NP cell proliferation and cell cycle by Notch signaling. For this, several key signaling pathways associated with cell growth, including PI3K/Akt and Wnt/β-catenin, were assessed. The results showed a significant inductive effect on the expression of PI3K/Akt and Wnt/β-catenin by JAG2 treatment, which was inhibited by Notch2/Heys2/Hes1 siRNA. This indicated that the PI3K/Akt and Wnt/β-catenin signaling pathways might take part in the Notch-mediated effect on proliferation in NP cells (Fig. 3c–f).

Then, we blocked the PI3K/Akt and Wnt/β-catenin pathways using the specific inhibitors LY294002 (10 μm) and XAV-939 (10 μm), and the results indicated that blocking of the PI3K/Akt and Wnt/β-catenin pathways led to a decrease in cyclin D1 and CDK6 expression. The elevated expression levels of cyclin D1 and CDK6 mRNA levels induced by Notch were abrogated by LY294002 and XAV-939 treatment alone or in combination (Fig. 3g, h). Western blot analysis showed similar results (Fig. 3i–k).

### JAG2/Notch2 signaling regulates TNF-α-induced apoptosis in NP cells

**Fig. 4:**
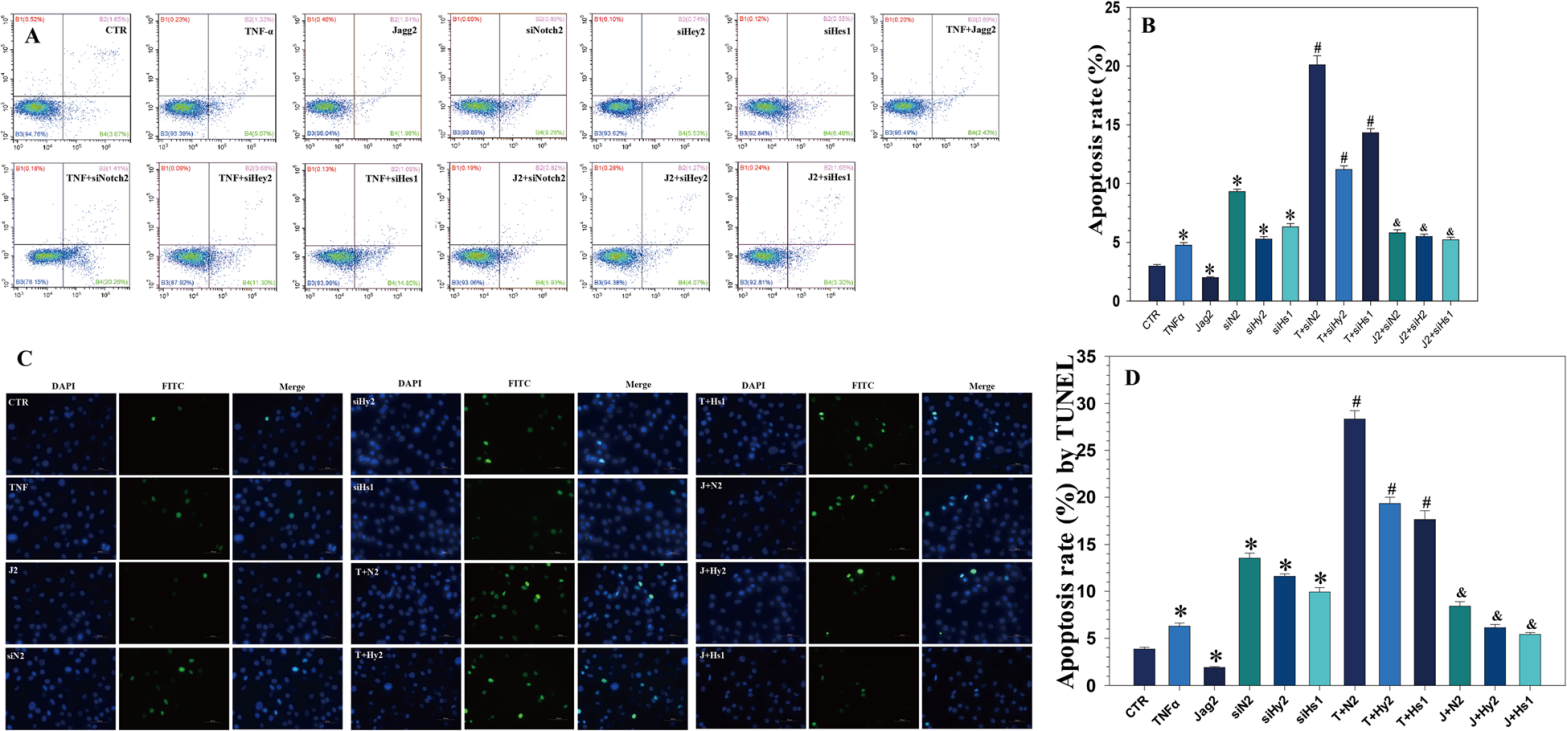
JAG2/Notch2 signaling inhibited TNF-α-induced apoptosis in NP cells. **a**, **b** TNF-α treatment increased the apoptotic rate of NP cells, which was reversed by recombinant JAG2 treatment; Notch2/Hey2/Hes1 siRNA significantly enhanced the apoptotic rate induced by TNF-α treatment. **c**, **d** TUNEL assay showed that TNF-α increased the apoptotic rate of NP cells, which was reversed by JAG2 treatment; Notch2/Hey2/Hes1 siRNA enhanced the apoptotic rate induced by TNF-α. *P* values were computed vs. non-stimulated controls*, TNF-α-stimulated controls^#^, or JAG2-stimulated controls^&^; *^,#,&^*P* < 0.05

NP cell apoptosis is considered a major cause of IVDD [4]. To investigate the effect of JAG2/Notch2 signaling on NP cell apoptosis, NP cells were treated with TNF-α, together with recombinant JAG2 and/or Notch2/Hey2/Hes1 siRNA. FITC-Annexin V/PI FCM assays were performed to assess the rate of NP cell apoptosis. After TNF-α treatment, the NP cell apoptotic rate increased, an effect that was reversed in the presence of recombinant JAG2. Notch2/Hey2/Hes1 siRNA increased the NP cell apoptotic rate, and they also increased NP cell apoptosis induced by TNF-α. Moreover, Notch2/Hes1/Hey2 siRNA suppressed the protective effect of recombinant JAG2 (Fig. 4a, b).

**Fig. 5:**
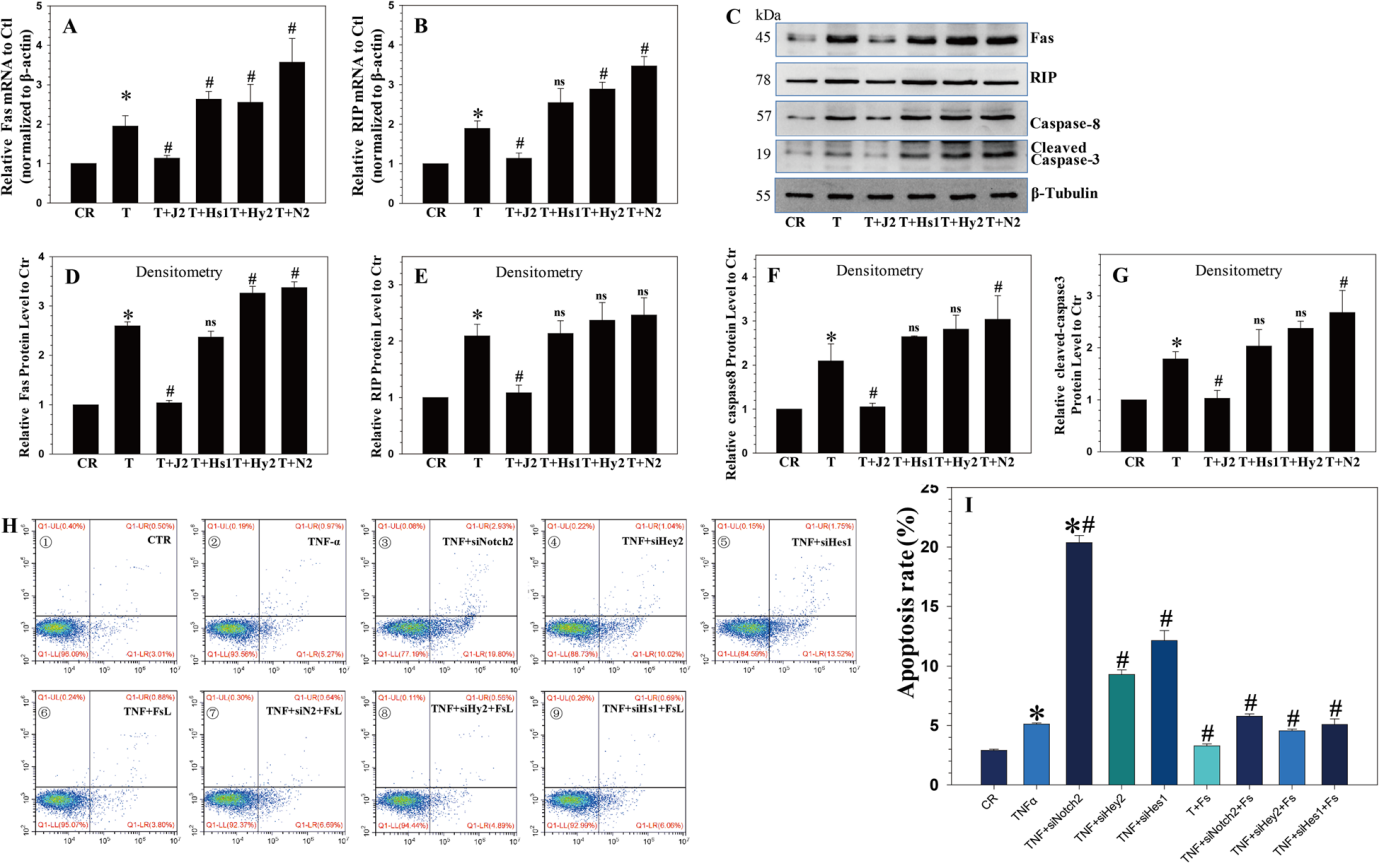
Notch inhibited TNF-α-promoted apoptosis via Fas. **a**, **b** Fas and RIP mRNA expression levels were upregulated by TNF-α stimulation and downregulated by JAG2 treatment; TUNEL assay showed similar results. **c**–**g** Western blot analysis showed that Fas, RIP1, cleaved-caspase-8, and cleaved-caspase-3 protein expression levels were upregulated by TNF-α and downregulated by JAG2 treatment; Notch2/Hes1/Hey2 siRNA significantly enhanced the induction by TNF-α. **h**, **i** The apoptotic mortality of cultured NP cells caused by TNF-α with or without Notch2/Hey2/Hes1 siRNA treatment was reduced by the addition of anti-FasL antibody to the culture. *P* values were computed vs. non-stimulated controls*, TNF-α-stimulated controls^#^, or JAG2-stimulated controls^&^; *^,#,&^*P* < 0.05

TUNEL staining demonstrated that TNF-α significantly induced NP cell apoptosis compared with that in the controls. Notch2/Hey2/Hes1 siRNA increased NP cell apoptosis, and they also enhanced NP cell apoptosis induced by TNF-α (Fig. 4c and 5a). These results are similar to the flow cytometry results, indicating that Notch signaling plays an important role in the regulation of NP cell apoptosis.

### Notch inhibits TNF-α-promoted apoptosis via suppressing the formation of the RIP1-FADD-caspase-8 complex

A previous study demonstrating the formation of the death-inducing signaling complex (DISC) was initiated by the activation of RIP1 trigger apoptosis following TNF-α induction [29]. To determine whether RIP1 participates in the TNF-α promotion of apoptosis in NP cells, they were treated with JAG2 or without Notch2/Hes1/Hey2 siRNA. As shown in Fig. 5b, c, Fas and RIP mRNA expression levels were upregulated by TNF-α stimulation. In contrast, the expression of these genes decreased in the JAG2 treatment group. Notch2/Hey2/Hes1 siRNA significantly enhanced the TNF-α-induced upregulation of Fas and RIP mRNA expression. Furthermore, Western blot analysis showed that Fas, RIP1, cleaved-caspase-8, and cleaved-caspase-3 protein expression levels were upregulated by TNF-α stimulation and downregulated by JAG2 treatment. Notch2/Hes1/Hey2 siRNA significantly enhanced the TNF-α-induced upregulation of Fas, RIP1, caspase-8, and cleaved-caspase-3 expression (Fig. 5d–g).

To determine whether the Fas/FasL system participated in the effect of Notch signaling on TNF-α-induced apoptosis, NP cells were treated with anti-FasL antibody before the TNF-α treatment with or without JAG2 or Notch2/Hey2/Hes1 siRNA. The results showed that the apoptotic mortality of cultured NP cells caused by TNF-α with or without Notch2/Hey2/Hes1 siRNA treatment was reduced by the addition of anti-FasL antibody to the culture (Fig. 5h–i).

**Fig. 6:**
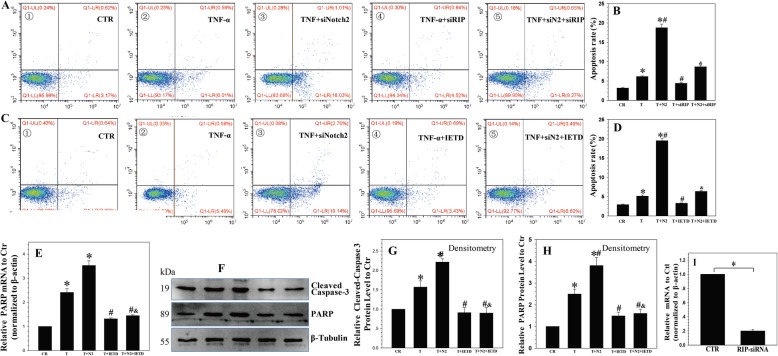
Notch inhibited TNF-α-promoted apoptosis via the RIP1-FADD-caspase-8 complex. **a**, **b** The apoptotic rate of NP cells treated with Notch2 siRNA plus TNF-α was decreased by the silencing of RIP. **c**, **d** z-IETD-fmk inhibited Notch2 siRNA-promoted cell death and apoptosis. **e-h** Real-time PCR and Western blotting showed that cleaved-caspase-3 and its substrate PARP were inhibited in Notch2 siRNA plus TNF-α-treated NP cells in the presence of z-IETD-fmk. **i** The efficiency of the RIP siRNA. *P* values were computed vs. non-stimulated controls*, TNF-α-stimulated controls^#^, or TNF-α and Notch2 siRNA-stimulated controls^&^; *^,#,&^*P* < 0.05

To confirm the important role of RIP1, a specific siRNA for RIP1 was transfected into NP cells; the transfection efficiency is shown in Fig. 6i. We found that the apoptotic rate of NP cells treated with Notch2 siRNA plus TNF-α was decreased by the knockdown of RIP1 (Fig. 6a, b).

Because caspase-8 is the effector of the RIP1-FADD-caspase-8 complex, which is responsible for cleaving downstream substrates [30], we speculated that caspase-8 acted as the initiator caspase in Notch2 siRNA-promoted apoptosis. To confirm this hypothesis, NP cells were treated with Notch2 siRNA and TNF-α in the presence of z-IETD-fmk, which is a caspase-8-specific inhibitor [31]. The results showed that the presence of z-IETD-fmk inhibited the Notch2 siRNA promotion of cell death and apoptosis (Fig. 6c, d) induced by TNF-α, confirming the significance of caspase-8 in Notch2 siRNA-promoted apoptosis. Notably, the activation of downstream caspase-3 and its substrate PARP was inhibited in Notch2 siRNA plus TNF-α-treated NP cells in the presence of z-IETD-fmk (Fig. 6e–h). These findings indicated that the Notch2 siRNA promotion of TNF-α-induced apoptosis in NP cells is dependent on the formation of the RIP1-FADD-caspase-8 complex and the subsequent activation of caspase-8.

### The role of JAG2/Notch2 in the regulation of IVDD in vivo

**Fig. 7:**
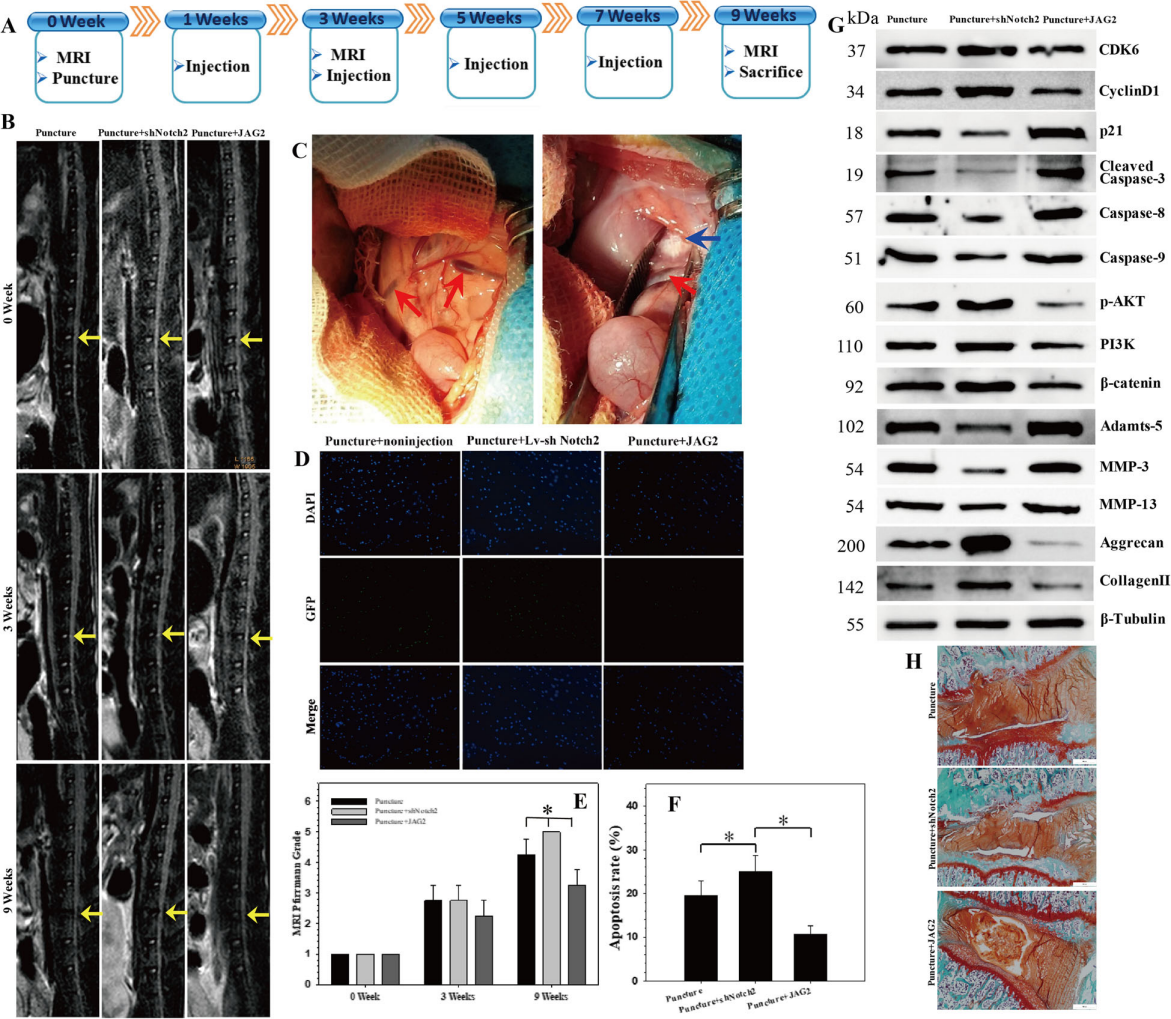
The role of JAG2/Notch2 in the regulation of IVDD in vivo. **a** A flow diagram of the in vivo experiments. A total of 32 rats were randomly divided into 4 groups: normal control, non-injection with puncture group (non-injection), recombinant JAG2 injection with puncture group (JAG2), and lentiviral sh-Notch2 injection with puncture group (sh-Notch2). **b** MRIs of the indicated groups were obtained 0/3/9 weeks after needle puncture. L4/5 (yellow arrow) was punctured, and the others were left intact. **c** Intraoperative anatomical images: the red arrow points to the renal iliolumbar vein, and the blue arrow points to the L4/5 intervertebral disc, which is a little higher than the iliolumbar vein. **d** TUNEL staining of the IVDs in the indicated groups at 9 weeks after needle puncture. DAPI indicating total cells; green fluorescence indicating TUNEL-positive cells. Scale bar = 100 μm. **e** The MRI grade in the indicated groups at 9 weeks after needle puncture. The degree of disc degeneration according to MRI grade was significantly lower in the JAG2 group than in the non-injection group; *P < 0.05. **f** A significant decrease in the apoptosis rate was noted in the JAG2 group compared with that in the puncture group, and there was an increased apoptosis rate in sh-Notch2 group compared with that in the puncture group; *P < 0.05. **g** Western blot analysis of the expression of CDK6, cyclin D1, p21, apoptotic effector caspases (caspase-3, caspase-8, and caspase-9), PI3K/Akt and Wnt/β-catenin signaling markers, catabolic enzymes (MMP-3, MMP-13, and Adamts-5), and extracellular matrix (ECM) components (collagen II, aggrecan) in the rat NP tissues. The injection of JAG2 alleviated the degenerative changes in the NP such as an enhanced apoptotic and catabolic response and reduced the expression of ECM components in the rat model of IVDD, while sh-Notch2 aggravated the degenerative changes in the NP. **h** Safranin O/fast green staining of the IVDs at 9 weeks after needle puncture showed signifcant IVDD in the sh-Notch2 group, while injection of JAG2 alleviated the degeneration of IVD

We successfully established a rat model of IVDD by needle puncture (Fig. 7a). MR images were recorded after disc puncture and recombinant JAG2 or lentiviral sh-Notch2 intradiscal injection. At 9 weeks after injection, the MRI degeneration score of the IVDs was significantly lower in the recombinant JAG2 group than in the non-injection group, while there was significantly increased disc degeneration (decreased signal intensity) in the lentiviral sh-Notch2 injection group compared with that in the non-injection group (Fig. 7b, c). The results of our MRI-guided quantitative evaluation, which was performed in accordance with the Pfirrmann grading system, confirmed that Notch signaling has protective effects on IVDD (Fig. 7e).

The injection of recombinant JAG2 alleviated the degenerative changes in the NP, such as a decreased apoptotic and catabolic response and increased expression of ECM components, in a rat model of IVDD. However, the injection of lentiviral sh-Notch2 aggravated the degenerative changes in the NP, such as an enhanced apoptotic and catabolic response and decrease in ECM contents (Fig. 7d–g). Safranin O/fast green staining of the IVDs indicated significant IVDD in the sh-Notch2 group, while injection of JAG2 alleviated the degeneration of IVD (Fig. 7h).

## Discussion

Regarding the role of Notch signaling in cell proliferation, previous studies have demonstrated that the effect of Notch on cell proliferation was cell specific [32]. Notch1 regulates the adipogenesis process, including the proliferation and differentiation of the adipocyte progenitor cells in vitro [33]. Dll4-Notch1 from the chamber endocardium can regulate cardiomyocyte proliferation and differentiation in a non-cell autonomous fashion [34]. The Notch inhibitor PF-03084014 was found to inhibit the self-renewal and proliferation of hepato-cellular carcinoma stem cells [35]. Overexpression of Dll3 in Lewis lung carcinoma cells promoted cell proliferation and reduced apoptosis in vitro [36]. Here, we determined the effect of the regulation of the Notch pathway on NP cell proliferation. We found that Notch signaling regulates NP cell proliferation through the JAG2/Notch2 axis, cyclin D1 is also critical for the Notch-mediated effect on proliferation, and JAG2 induced the proliferation of NP cells, while the depletion of Notch2/Hey2/Hes1 reduced the proliferation of NP cells.

To test the premise that Notch signaling regulates NP cell proliferation by modulating the cell cycle, we investigated several known G0/G1 cell cycle phase regulatory factors. We found that consistent with the cell cycle regulation, cyclin D1 and CDK2 protein expression levels were increased by recombinant JAG2 treatment and decreased by silencing of Notch2, Hes1, or Hey2, while p21 protein expression was decreased by recombinant JAG2 treatment and increased by silencing of Notch2, Hes1, or Hey2. These results are similar to those of previous studies; Zhang et al. [37]. found that Notch activation promoted the cell cycle in proliferating hepatocytes, and Qu et al. [38] found that the inhibition of Notch signaling increased basophil apoptosis and suppressed basophil proliferation and that inhibition of Notch signaling reduced basophils in the S phase, with concomitant accumulation in G1 and G2 phases. However, other studies reported opposite results in other cells; Sun et al. [39] found that the overexpression of the active intracellular domain of all four Notch receptors (ICN1–4) suppressed cervical cancer Hela cell growth, with ICN1 inducing cell cycle arrest in phase G1. The overexpression of NICD in chondrocytes was found to enhance the G0/G1 cell cycle transition and cell cycle arrest, together with decreased expression of a cell cycle inhibitor (p57, [40]). A Notch inhibitor-induced G2/M cell cycle arrest through the downregulation of the expression of cyclin D, cyclin E1, cyclin E2, and pRb and the upregulation of P21, P27, and P53 expression in nasopharyngeal carcinoma cells [41]. These data suggest that Notch-induced cellular phenotype is likely to be specific and mediated by activation of the Notch receptors.

Among the multiple factors that are involved in controlling cell proliferation, here, we found that Notch signaling activated the PI3K/Akt and Wnt/β-catenin pathways in NP cells. Notably, the anti-apoptotic and proliferation- and differentiation-promoting effects of Notch signaling have been demonstrated to depend on the PI3K/Akt pathways [42]. Sangphech et al. [43] also reported that Notch signaling positively regulates the phosphorylation of Akt to affect the survival and cell cycle of macrophages following LPS stimulation. The classic Wnt/β-catenin signaling pathway is also known to play a critical role in cell proliferation and homeostasis. Here, we found that the PI3K/Akt and Wnt/β-catenin signaling pathways may take part in Notch-mediated proliferation in NP cells.

Apoptosis is known to be responsible for IVD degeneration, and the Notch gene suppresses apoptosis through a growth factor-mediated survival pathway [44]. We, therefore, conducted flow cytometry assays to determine if the alteration of Notch signaling affected the cell apoptosis. Our results indicated that recombinant JAG2 reversed the NP cell apoptosis induced by TNF-α, while Notch2/Hes1/Hey2 siRNA significantly enhanced the apoptotic rate induced by TNF-α treatment. These results suggested that cell apoptosis contributes to IVD degeneration through Notch signaling, which is similar to the finding of a previous study. Zou et al. [45] reported that Notch2 is important for anti-apoptosis and metastasis in laryngeal squamous cell carcinoma, and Notch2 knockdown decreased cell proliferation and increased cell apoptosis in Hep-2 cells. Chen et al. [46] also demonstrated that Notch-1 knockdown was associated with a significantly higher proportion of late apoptotic and necrotic cells in salivary adenoid cystic carcinoma. Yu and Song [47] found that Notch-1 signaling reduced cardiomyocyte apoptosis through regulating Bcl-2, Bax, and caspase-9/3. Murata et al. [48] demonstrated that Notch activation induced by the recombinant delta-1 reduced the TNF-alpha-induced growth suppression and apoptosis in U937 cells.

In this study, we found that Notch activation induced by recombinant JAG2 reduced the TNF-α-induced growth suppression and apoptosis in NP cells. Previous studies demonstrated that the formation of the DISC was initiated by the activation of RIP1 trigger apoptosis following TNF-α induction [29]. In cell lines sensitive to TNF-α-induced apoptosis, it has been shown that two types of signaling complexes form following TNF-α exposure. One forms at the cell surface and involves the interaction of TNFR1 with TRADD, RIP1, and TRAF2, and the second is devoid of TNFR1 but engages FADD and caspase-8 [49]. Therefore, we thought that JAG2 stimulation might reduce the expression of the Fas-associated death domain (FADD) adaptor molecule. We found JAG2 treatment decreased the expression of Fas, RIP1, caspase-8, and cleaved-caspase-3, while Notch2/Hey2/Hes1 siRNA significantly enhanced the TNF-α-induced upregulation of Fas, RIP1, caspase-8, and cleaved-caspase-3 expression. According to Eum et al. [50], TRADD and RIP bind to the FADD adaptor molecule, resulting in caspase-8 recruitment in the cytosol. The interaction between FADD and pro-caspase-8 leads to apoptosis through protein cleavage and activation of the downstream caspase cascade, as well as the cleavage and activation of pro-apoptotic targets.

## Conclusions

**Fig. 8:**
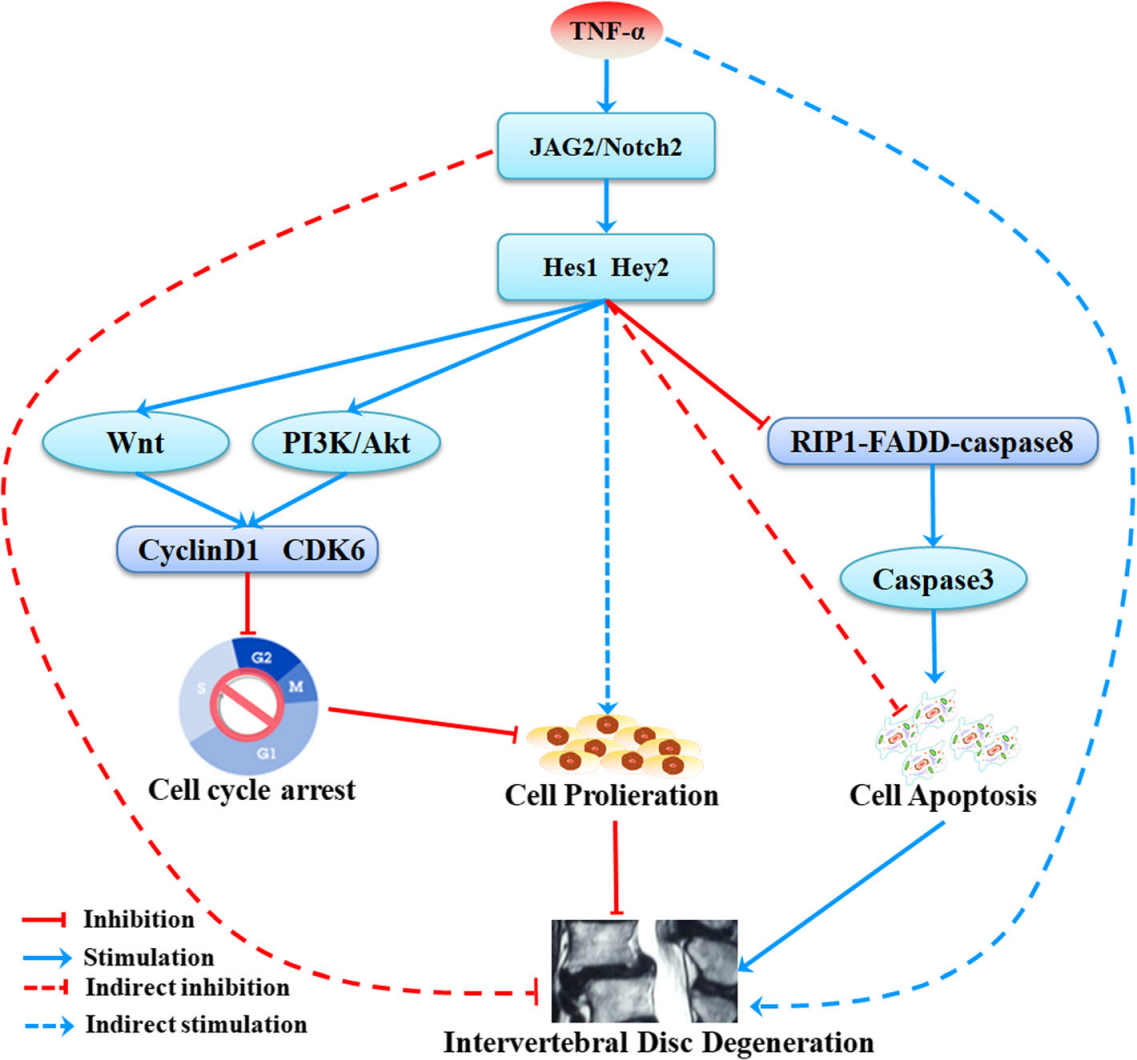
Summary of the findings and proposed mechanisms. Our data indicated that JAG2/Notch2 inhibited IVDD by modulating cell proliferation, apoptosis, and extracellular matrix. The JAG2/Notch2 axis induced NP cell proliferation via PI3K/Akt and Wnt/β-catenin signaling and inhibited TNF-α-induced apoptosis by suppressing the formation of the RIP1-FADD-caspase-8 complex. In conclusion, JAG2/Notch2 inhibited IVDD by modulating cell proliferation, apoptosis, and extracellular matrix

In summary, our data represent the first evidence that Notch signaling plays an important role in the processes of NP cell proliferation and apoptosis. We demonstrated that the JAG2/Notch2 axis regulates NP cell proliferation and apoptosis via PI3K/Akt and Wnt/β-catenin signaling. Moreover, Notch signaling can inhibit TNF-α-induced apoptosis via suppressing the formation of the RIP1-FADD-caspase-8 complex. Based on these findings and those of previous studies, we propose that the activation of Notch signaling can delay IVDD progression and maintain the normal function of IVDs (Fig. 8). Current and previous research shed light on the therapeutic implications of targeting the JAG2/Notch2 axis to inhibit or reverse IVDD. These results will aid in the future development of a drug that targets the increased expression of the JAG2/Notch2 axis to inhibit or reverse IVDD, thus reducing the incidence of LBP.

## Supplementary information

**Additional file 1:** IHC of the expression levels of JAG2 and Notch-2 in normal and degenerated IVDs.

**Additional file 2:** IHC of negative control to show staining of JAG2 and Notch-2 in normal and degenerated IVDs.

**Additional file 3:** The transfection efficiency of Notch2/Hes1/Hey2 siRNA in NPCs.

## Data Availability

All data supporting our findings are contained within the manuscript.

## Abbreviations

CCK-8: Cell Counting Kit-8
DMEM: Dulbecco’s modified Eagle’s medium
DISC: Death-inducing signaling complex
FBS: Fetal bovine serum
FCM: Flow cytometric
ECM: Extracellular matrix
IVDD: Intervertebral disc degeneration
LBP: Low back pain
NP: Nucleus pulposus
OD: Optical density
qRT-PCR: Quantitative reverse transcription polymerase chain reaction
TUNEL: Terminal deoxynucleotidyl transferase (TdT)-mediated dUTP nick end labeling

## References

[1] van Dieen JH, Kuijer PP, Burdorf A, Marras WS, Adams MA (2012). Non-specific low back pain. Lancet..

[2] Wang X, Wang H, Yang H, Li J, Cai Q, Shapiro IM (2014). Tumor necrosis factor-alpha- and interleukin-1beta-dependent matrix metalloproteinase-3 expression in nucleus pulposus cells requires cooperative signaling via syndecan 4 and mitogen-activated protein kinase-NF-kappaB axis: implications in inflammatory disc disease. Am J Pathol.

[3] Livshits G, Popham M, Malkin I, Sambrook PN, Macgregor AJ, Spector T (2011). Lumbar disc degeneration and genetic factors are the main risk factors for low back pain in women: the UK Twin Spine Study. Ann Rheum Dis.

[4] Wang H, Liu H, Zheng ZM, Zhang KB, Wang TP, Sribastav SS (2011). Role of death receptor, mitochondrial and endoplasmic reticulum pathways in different stages of degenerative human lumbar disc. Apoptosis.

[5] Wang SZ, Rui YF, Lu J, Wang C (2014). Cell and molecular biology of intervertebral disc degeneration: current understanding and implications for potential therapeutic strategies. Cell Prolif.

[6] Ding F, Shao ZW, Xiong LM (2013). Cell death in intervertebral disc degeneration. Apoptosis.

[7] Jiang L, Zhang X, Zheng X, Ru A, Ni X, Wu Y (2013). Apoptosis, senescence, and autophagy in rat nucleus pulposus cells: implications for diabetic intervertebral disc degeneration. J Orthop Res.

[8] Roberts S, Evans H, Trivedi J, Menage J (2006). Histology and pathology of the human intervertebral disc. J Bone Joint Surg Am.

[9] Hoyland JA, Le Maitre C, Freemont AJ (2008). Investigation of the role of IL-1 and TNF in matrix degradation in the intervertebral disc. Rheumatology (Oxford).

[10] Pockert AJ, Richardson SM, Le Maitre CL, Lyon M, Deakin JA, Buttle DJ (2009). Modified expression of the ADAMTS enzymes and tissue inhibitor of metalloproteinases 3 during human intervertebral disc degeneration. Arthritis Rheum.

[11] Wang J, Chen H, Cao P, Wu X, Zang F, Shi L (2016). Inflammatory cytokines induce caveolin-1/beta-catenin signalling in rat nucleus pulposus cell apoptosis through the p38 MAPK pathway. Cell Prolif.

[12] Hadjipavlou AG, Tzermiadianos MN, Bogduk N, Zindrick MR (2008). The pathophysiology of disc degeneration: a critical review. J Bone Joint Surg Br.

[13] Hiyama A, Yokoyama K, Nukaga T, Sakai D, Mochida J (2013). A complex interaction between Wnt signaling and TNF-alpha in nucleus pulposus cells. Arthritis Res Ther.

[14] Richards GS, Degnan BM (2009). The dawn of developmental signaling in the metazoa. Cold Spring Harb Symp Quant Biol.

[15] Siebel C, Lendahl U (2017). Notch signaling in development, tissue homeostasis, and disease. Physiol Rev.

[16] Haller R, Schwanbeck R, Martini S, Bernoth K, Kramer J, Just U (2012). Notch1 signaling regulates chondrogenic lineage determination through Sox9 activation. Cell Death Differ.

[17] Watanabe N, Tezuka Y, Matsuno K, Miyatani S, Morimura N, Yasuda M (2003). Suppression of differentiation and proliferation of early chondrogenic cells by Notch. J Bone Miner Metab.

[18] Hiyama A, Skubutyte R, Markova D, Anderson DG, Yadla S, Sakai D (2011). Hypoxia activates the notch signaling pathway in cells of the intervertebral disc: implications in degenerative disc disease. Arthritis Rheum.

[19] Wang H, Tian Y, Wang J, Phillips KL, Binch AL, Dunn S (2013). Inflammatory cytokines induce NOTCH signaling in nucleus pulposus cells: implications in intervertebral disc degeneration. J Biol Chem.

[20] Ye F, Wang H, Zheng Z, He P, Sribastav SS, Wang H (2017). Role of SHOX2 in the development of intervertebral disc degeneration. J Orthop Res.

[21] Li Z, Liu H, Yang H, Wang J, Wang H, Zhang K (2014). Both expression of cytokines and posterior annulus fibrosus rupture are essential for pain behavior changes induced by degenerative intervertebral disc: an experimental study in rats. J Orthop Res.

[22] Zhang J, Li Z, Chen F, Liu H, Wang H, Li X (2017). TGF-beta1 suppresses CCL3/4 expression through the ERK signaling pathway and inhibits intervertebral disc degeneration and inflammation-related pain in a rat model. Exp Mol Med.

[23] Pfirrmann CW, Metzdorf A, Zanetti M, Hodler J, Boos N (2001). Magnetic resonance classification of lumbar intervertebral disc degeneration. Spine..

[24] Masuda K, Aota Y, Muehleman C, Imai Y, Okuma M, Thonar EJ (2005). A novel rabbit model of mild, reproducible disc degeneration by an anulus needle puncture: correlation between the degree of disc injury and radiological and histological appearances of disc degeneration. Spine..

[25] Berenstein R, Nogai A, Waechter M, Blau O, Kuehnel A, Schmidt-Hieber M (2016). Multiple myeloma cells modify VEGF/IL-6 levels and osteogenic potential of bone marrow stromal cells via Notch/miR-223. Mol Carcinog.

[26] Gopalakrishnan N, Saravanakumar M, Madankumar P, Thiyagu M, Devaraj H (2014). Colocalization of β-catenin with Notch intracellular domain in colon cancer: a possible role of Notch1 signaling in activation of CyclinD1-mediated cell proliferation. Mol Cell Biochem.

[27] Velicky P, Haider S, Otti GR, Fiala C, Pollheimer J, Knofler M (2014). Notch-dependent RBPJκ inhibits proliferation of human cytotrophoblasts and their differentiation into extravillous trophoblasts. Mol Hum Reprod.

[28] Munnamalai V, Fekete DM (2016). Notch-Wnt-Bmp crosstalk regulates radial patterning in the mouse cochlea in a spatiotemporal manner. Development.

[29] Zheng M, Wu Z, Wu A, Huang Z, He N, Xie X (2016). MiR-145 promotes TNF-alpha-induced apoptosis by facilitating the formation of RIP1-FADDcaspase-8 complex in triple-negative breast cancer. Tumour Biol.

[30] Abhari BA, Cristofanon S, Kappler R, von Schweinitz D, Humphreys R, Fulda S (2013). RIP1 is required for IAP inhibitor-mediated sensitization for TRAIL-induced apoptosis via a RIP1/FADD/caspase-8 cell death complex. Oncogene..

[31] Roberge S, Roussel J, Andersson DC, Meli AC, Vidal B, Blandel F (2014). TNF-alpha-mediated caspase-8 activation induces ROS production and TRPM2 activation in adult ventricular myocytes. Cardiovasc Res.

[32] Demitrack ES, Samuelson LC (2017). Notch as a driver of gastric epithelial cell proliferation. Cell Mol Gastroenterol Hepatol.

[33] Shan T, Liu J, Wu W, Xu Z, Wang Y (2017). Roles of notch signaling in adipocyte progenitor cells and mature adipocytes. J Cell Physiol.

[34] D’Amato G, Luxan G, de la Pompa JL (2016). Notch signalling in ventricular chamber development and cardiomyopathy. FEBS J.

[35] Wu CX, Xu A, Zhang CC, Olson P, Chen L, Lee TK, et al. Notch inhibitor PF-03084014 inhibits hepatocellular carcinoma growth and metastasis via suppression of cancer stemness due to reduced activation of Notch1-Stat3. Mol Cancer Ther. 2017;16(8):1531-1543. 10.1158/1535-7163.MCT-17-0001 28522590

[36] Deng SM, Yan XC, Liang L, Wang L, Liu Y, Duan JL (2017). The Notch ligand delta-like 3 promotes tumor growth and inhibits Notch signaling in lung cancer cells in mice. Biochem Biophys Res Commun.

[37] Zhang F, Zhang J, Li X, Li B, Tao K, The YS. Notch signaling pathway regulates cell cycle in proliferating hepatocytes involved in liver regeneration. J Gastroenterol Hepatol. 2018;33(8):1538-1547. 10.1111/jgh.14110 29384233

[38] Qu SY, Lin JJ, Zhang J, Song LQ, Yang XM, Wu CG (2017). Notch signaling pathway regulates the growth and the expression of inflammatory cytokines in mouse basophils. Cell Immunol.

[39] Sun L, Liu M, Sun GC, Yang X, Qian Q, Feng S (2016). Notch signaling activation in cervical cancer cells induces cell growth arrest with the involvement of the nuclear receptor NR4A2. J Cancer.

[40] Shang X, Wang J, Luo Z, Wang Y, Morandi MM, Marymont JV (2016). Notch signaling indirectly promotes chondrocyte hypertrophy via regulation of BMP signaling and cell cycle arrest. Sci Rep.

[41] Chen SM, Liu JP, Zhou JX, Chen C, Deng YQ, Wang Y (2011). Suppression of the notch signaling pathway by gamma-secretase inhibitor GSI inhibits human nasopharyngeal carcinoma cell proliferation. Cancer Lett.

[42] Meurette O, Stylianou S, Rock R, Collu GM, Gilmore AP, Brennan K (2009). Notch activation induces Akt signaling via an autocrine loop to prevent apoptosis in breast epithelial cells. Cancer Res.

[43] Sangphech N, Osborne BA, Palaga T (2014). Notch signaling regulates the phosphorylation of Akt and survival of lipopolysaccharide-activated macrophages via regulator of G protein signaling 19 (RGS19). Immunobiology..

[44] Helbig C, Gentek R, Backer RA, de Souza Y, Derks IA, Eldering E (2012). Notch controls the magnitude of T helper cell responses by promoting cellular longevity. Proc Natl Acad Sci U S A.

[45] Zou Y, Fang F, Ding YJ, Dai MY, Yi X, Chen C (2016). Notch 2 signaling contributes to cell growth, anti-apoptosis and metastasis in laryngeal squamous cell carcinoma. Mol Med Rep.

[46] Chen W, Cao G, Yuan X, Zhang X, Zhang Q, Zhu Y (2015). Notch-1 knockdown suppresses proliferation, migration and metastasis of salivary adenoid cystic carcinoma cells. J Transl Med.

[47] Yu B, Song B (2014). Notch 1 signalling inhibits cardiomyocyte apoptosis in ischaemic postconditioning. Heart Lung Circ.

[48] Murata-Ohsawa M, Tohda S, Kogoshi H, Nara N (2004). The Notch ligand, Delta-1, reduces TNF-alpha-induced growth suppression and apoptosis by decreasing activation of caspases in U937 cells. Int J Mol Med.

[49] Muppidi JR, Tschopp J, Siegel RM (2004). Life and death decisions: secondary complexes and lipid rafts in TNF receptor family signal transduction. Immunity..

[50] Eum HA, Vallabhaneni R, Wang Y, Loughran PA, Stolz DB, Billiar TR (2011). Characterization of DISC formation and TNFR1 translocation to mitochondria in TNF-alpha-treated hepatocytes. Am J Pathol.

