# Supplementary figures and images for: JAG2/Notch2 inhibits intervertebral disc degeneration by modulating cell proliferation, apoptosis, and extracellular matrix
Source: Arthritis Res Ther. 2019 Oct 16;21:213. doi: 10.1186/s13075-019-1990-z (PMC6796488; doi:10.1186/s13075-019-1990-z)

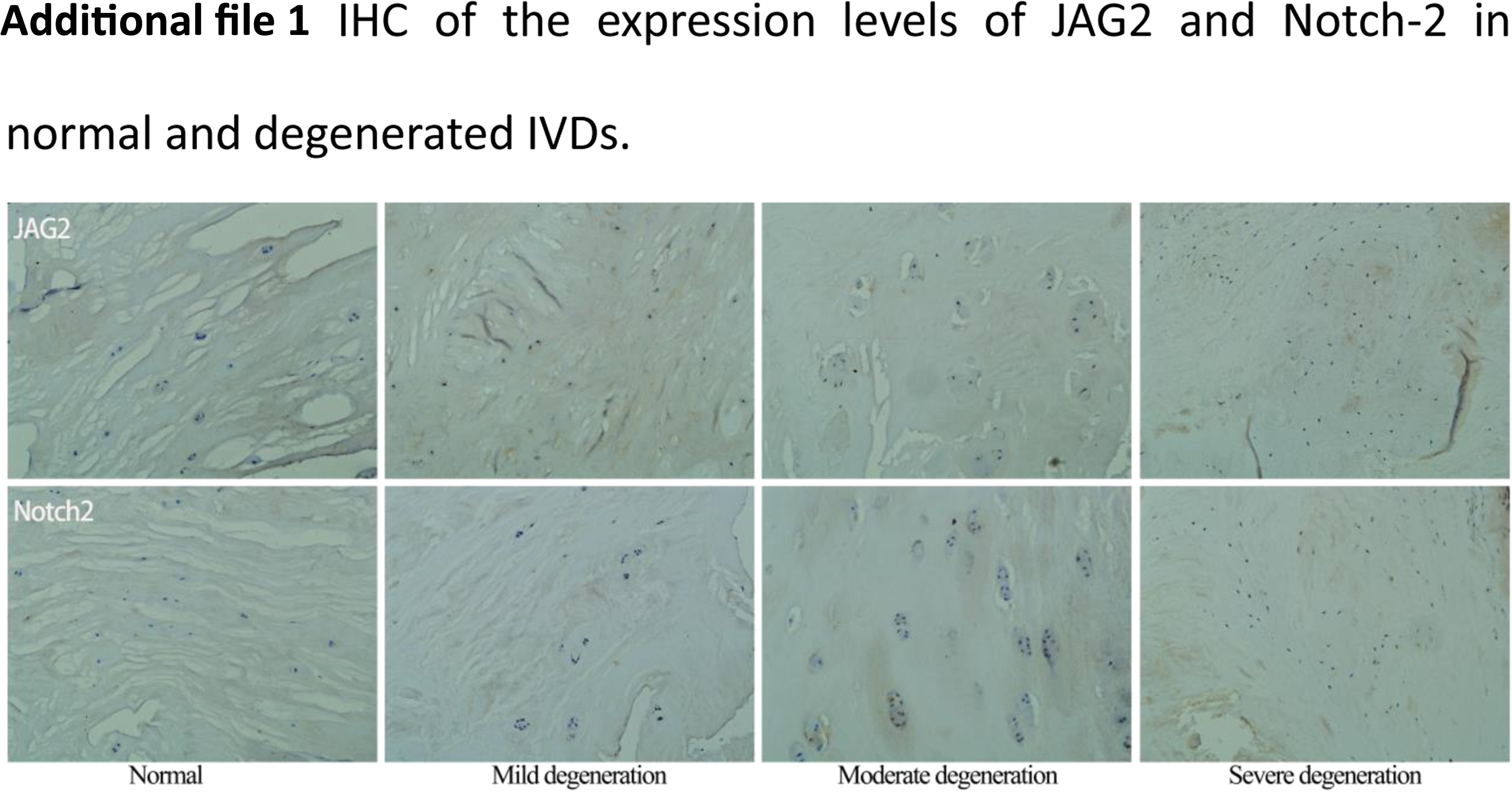

Supplement: Supplementary file 1 — Additional file 1: IHC of the expression levels of JAG2 and Notch-2 in normal and degenerated IVDs. [file 13075_2019_1990_MOESM1_ESM.tif]

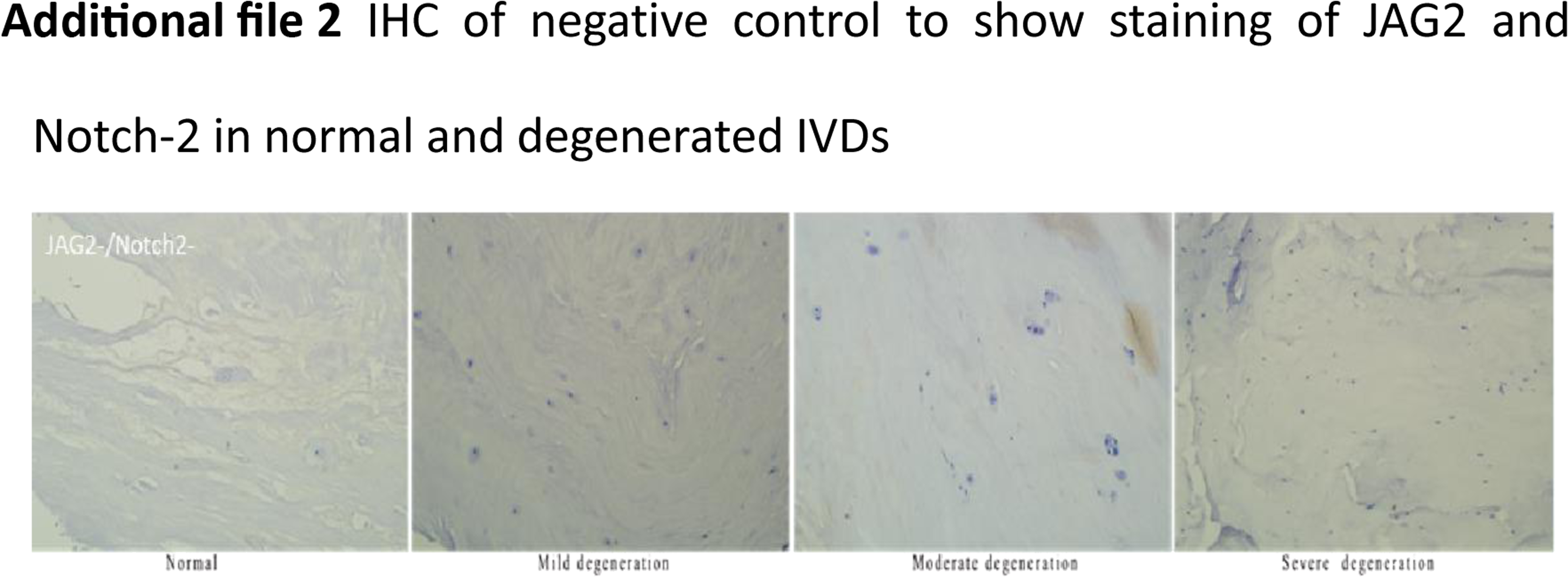

Supplement: Supplementary file 2 — Additional file 2: IHC of negative control to show staining of JAG2 and Notch-2 in normal and degenerated IVDs. [file 13075_2019_1990_MOESM2_ESM.tif]

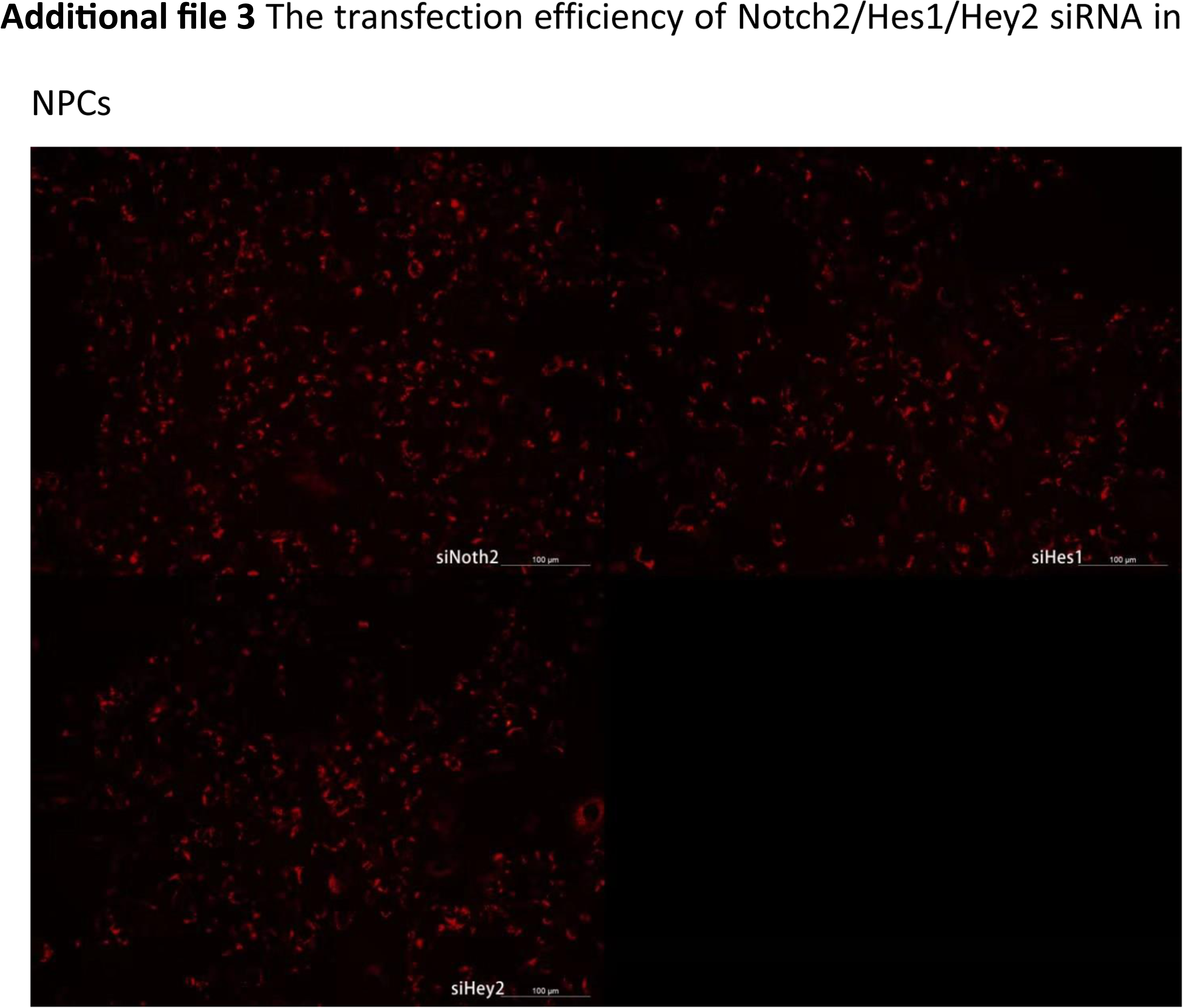

Supplement: Supplementary file 3 — Additional file 3: The transfection efficiency of Notch2/Hes1/Hey2 siRNA in NPCs. [file 13075_2019_1990_MOESM3_ESM.tif]
